# Transformer-Based Clinical Annotation of Lung Cancer Reports: A Benchmark and Fine-Tuning Study on a Novel Tunisian Corpus

**DOI:** 10.3390/bioengineering13070724

**Published:** 2026-06-24

**Authors:** Ranim Yahyaoui, Ismail Dergaa, Jean Noël Nikiema, Halil İbrahim Ceylan, Nicola Luigi Bragazzi, Saoussen Hantous-Zannad, Hanene Boussi Rahmouni

**Affiliations:** 1 Laboratory of Biophysics and Medical Technologies, Higher Institute of Medical Technologies of Tunis (ISTMT), University of Tunis El Manar, Tunis 1002, Tunisia; ranim.yahyaoui@etudiant-istmt.utm.tn (R.Y.); hanene.boussi@istmt.utm.tn (H.B.R.); 2 High Institute of Sport and Physical Education of Ksar Said, University of Manouba, Manouba 2010, Tunisia; phd.dergaa@gmail.com; 3 Physical Activity Research Unit, Sport and Health (UR18JS01), National Observatory of Sports, Tunis 1003, Tunisia; 4 High Institute of Sport and Physical Education of Kef, University of Jendouba, Jendouba 7100, Tunisia; 5 Department of Management, Evaluation and Health Policy, School of Public Health, University of Montreal, Montreal, QC H3T 1J4, Canada; jean.nikiema@umontreal.ca; 6 Physical Education of Sports Teaching Department, Faculty of Sports Sciences, Atatürk University, Erzurum 25240, Türkiye; 7 Department of Clinical Pharmacy, Saarland University, 66123 Saarbrücken, Germany; 8 Faculty of Medicine of Tunis, University of Tunis El Manar, Tunis 1007, Tunisia; saoussen.hantous@fmt.utm.tn; 9 Department of Radiology, Abderrahmane Mami Hospital, Ariana 2035, Tunisia; 10 The Computer Science Research Center, The University of the West of England, Bristol BS16 1QY, UK

**Keywords:** BioClinicalBERT, clinical NLP, DrBERT, lung cancer, named-entity recognition, NER, RoBERTa, TNM staging, Tunisian corpus, transformer models

## Abstract

**Background:** Lung cancer causes more deaths than any other malignancy worldwide, accounting for 2.2 million new cases and 1.8 million deaths in 2020. Extracting structured clinical knowledge from unstructured French-language oncology records remains methodologically unresolved in Tunisian and Francophone healthcare systems, where validated natural language processing tools do not yet exist. This study examined the effectiveness of transformer-based named-entity recognition for automated clinical annotation of Tunisian lung cancer reports. **Aim:** The study aimed to (i) establish performance baselines for four transformer-based models on a publicly available thoracic radiology dataset, (ii) evaluate five models, including a French biomedical specialist, on a newly constructed Tunisian clinical corpus, and (iii) demonstrate prototype deployment feasibility for structured clinical decision support. **Methods:** An initial comparative study evaluated BERT, RoBERTa, BioClinicalBERT, and CamemBERT using the official RadGraph dataset partitions, which natively comprise a total of 600 annotated thoracic radiology reports distributed across a standardized 80/10/10 split. Subsequently, five models were evaluated on 200 manually annotated diagnostic reports from Mami Pneumo-Phthisiology Hospital, Tunis. For the Tunisian corpus, a five-fold cross-validation approach was implemented to ensure robust performance estimation, followed by final evaluation on a dedicated hold-out test set. All models were trained for a maximum of 10 epochs, with a learning rate of 5 × 10^−5^ and a batch size of 16. **Results:** Based on the initial comparative study on the RadGraph dataset, where RoBERTa was the top performer and achieved the highest F1-score of 0.873 (precision: 0.869, recall: 0.877), we evaluated its specialized biomedical variant, DR-BERT, on our Tunisian clinical dataset. DR-BERT demonstrated strong generalization on the hold-out test set with an F1-score of 0.824, outperforming the baseline RoBERTa (test F1: 0.791) and showing competitive performance relative to multilingual BERT (0.843 ± 0.005 in five-fold cross-validation). A prototype interface generated structured clinical summaries encompassing prior conditions, imaging modalities, and TNM staging. Conclusion: Language- and domain-adapted transformer models effectively extract structured clinical entities from French-language Tunisian lung cancer reports. DR-BERT’s superior generalization on unseen data confirms that biomedical pretraining in the target language is a key driver of robust performance in specialized French oncology text. This work establishes foundational infrastructure for NLP-driven oncology data management in Tunisia and comparable Francophone settings.

## 1. Introduction

Lung cancer occupies a singular and devastating position in global oncology: it ranks second in incidence yet leads all malignancies in mortality across both sexes, accounting for 2.2 million new cases and 1.8 million deaths in 2020 alone [1]. The five-year survival rate in high-income countries remains at approximately 15%, a figure largely unchanged despite decades of therapeutic advancement [2]. Two converging factors explain this persistent lethality. The first is late-stage diagnosis: the overwhelming majority of patients present at stage III or IV, when curative resection is no longer feasible and systemic therapies offer limited durable benefit [3]. The second is the biological complexity of the disease itself. Lung cancer exhibits profound heterogeneity across imaging phenotypes, histopathological subtypes, genomic alterations, and protein expression profiles, rendering treatment selection—chemotherapy, targeted therapy, immunotherapy, or multimodal combinations with surgery and radiotherapy—inherently dependent on granular individual data [4,5]. Precision medicine is the conceptual and practical response to this challenge: matching therapeutic strategies to the individual patient’s molecular and clinical profile by integrating genomic, radiomic, and longitudinal clinical data [4,6].

However, a critical barrier prevents the large-scale implementation of precision medicine: the inability to systematically extract these granular data from the massive volume of unstructured clinical narratives generated in hospital workflows. Despite the richness of available information, there is a persistent research gap in developing automated tools capable of handling the linguistic specificities of non-English clinical reports, particularly in Francophone regions like Tunisia. This study addresses this problem by developing a specialized Natural Language Processing (NLP) framework to bridge the gap between narrative clinical text and structured oncological intelligence. Realizing this vision requires continuous, structured, and reliable access to patient information at scales that manual clinical workflows cannot sustain.

Electronic health records constitute the primary repository of clinical intelligence in contemporary healthcare systems. They contain longitudinal, multi-domain data encompassing patient demographics, laboratory values, imaging reports, pathology findings, disease progression notes, and treatment records [7,8]. A substantial and systematically underutilized proportion of this information, however, is embedded in free-text clinical narratives rather than structured database fields. Radiology reports, oncology consultation notes, and surgical pathology summaries document critical clinical reasoning in natural language, rendering them resistant to automated computational query and analysis [8,9]. The resulting gap between information stored in electronic health records and information extractable from them is one of the most consequential inefficiencies in contemporary clinical and translational research [10]. Manual information extraction is costly, time-consuming, prone to inter-rater inconsistency, and fundamentally unscalable at the data volumes required for population-level oncology research or real-world evidence generation [9,11]. Training staff on electronic health record platforms addresses workflow barriers but does not resolve the unstructured text problem; Musa et al. demonstrated in a Qatari wellness context that even targeted one-to-one training programs substantially reduced booking times and improved practical competency, yet the challenge of extracting structured knowledge from narrative clinical text remained entirely unaddressed [12]. Automated NLP has emerged as the methodological response, enabling systematic identification and extraction of clinically meaningful entities from free text, converting narrative documentation into structured, computable data at scale [7,11]. The clinical implications are substantial: structured NLP outputs can support patient stratification, epidemiological surveillance, real-world evidence generation, clinical trial recruitment, and construction of clinical decision support systems [13,14].

The methodological trajectory of clinical NLP has advanced through three identifiable phases, beginning with rule-based systems. This earliest generation typically follows a structured pipeline involving text tokenization, lexical analysis, standard concept mapping, and negator processing. These systems rely on handcrafted lexicons, regular expressions, and curated ontologies—such as the Unified Medical Language System (UMLS)—to identify clinical concepts.

For instance, Nguyen et al. [9,15] presented a symbolic rule-based methodology utilizing the UMLS annotator for TNM (Tumor, Nodes, and Metastases) classification in lung cancer pathology reports. Their system achieved classification accuracies of 72%, 78%, and 94% for the T, N, and M stages, respectively [15,16]. Similarly, Beyer et al. [16] employed a rule-based approach to capture imaging characteristics of lung nodules from structured CT reports and suggested the applicable Lung-RADS™ category. This algorithm successfully identified positive nodules with an overall sensitivity of 75.0% and a specificity of 98.8% [17]. Furthermore, the clinical Text Analysis and Knowledge Extraction System (cTAKES), developed by Savova et al. [18], integrated these rule-based foundations with machine learning components into a modular pipeline covering sentence segmentation, part-of-speech tagging, named-entity recognition, and negation detection. While subsequent machine learning approaches improved upon these performances, they remained constrained by the necessity for extensive and labor-intensive feature engineering [18,19].

In early research, Iain McCowan et al. investigated the classification of a patient’s lung cancer stage based on analysis of their free-text medical reports. The system uses NLP to transform the report text, including identification of UMLS terms and detection of negated findings. The transformed report is then classified using statistical machine learning techniques. A support vector machine (SVM) is trained for each stage category based on word occurrences in a corpus of histology reports for pathologically staged patients [20].

Machine learning-based methods are often utilized alongside rule-based approaches, demonstrating improved performance over using rule-based methods alone. However, they continue to face similar challenges that are inherent to rule-based techniques [21].

Deep learning addressed this constraint by automatically extracting features from raw text. Gupta et al. investigated Long Short-Term Memory networks for clinical entity extraction from CT text reports [15]; Chen et al. demonstrated that convolutional neural networks matched or exceeded traditional NLP models for pulmonary embolism classification in thoracic CT reports [18].

The most consequential shift came with transformer-based architectures. BERT [19,22] introduced a bidirectional contextual representation that substantially elevated performance across NLP tasks. Domain adaptation through targeted pretraining on biomedical and clinical corpora produced the current specialist generation: BioClinicalBERT, pretrained on MIMIC-III clinical notes [23], and DrBERT, a RoBERTa architecture pretrained on the French biomedical corpus NACHOS [24,25].

Machine learning methods more broadly have demonstrated strong classification performance across health contexts, with ensemble and deep learning models achieving 84–92.4% accuracy in predicting sedentary behavior from sensor data [26] and an AUROC of 0.98 in predicting early ARDS from local Tunisian clinical data [27]. These findings collectively establish that high-performing clinical AI systems can be built on regionally collected datasets with appropriate methodological choices. Abdaoui et al. demonstrated this directly in the Tunisian pathology context, where a hybrid BioClinicalBERT model augmented with dense retrieval achieved an F1-score of 0.97 for clinical entity extraction from locally collected pathology reports, confirming both the feasibility and the performance attainable with domain-adapted architectures on Tunisian clinical data [28].

This research builds upon these advancements to address the remaining structural gap. The overwhelming majority of clinical NLP research has been conducted in English, with more recent extensions to Chinese clinical narratives [29]. French-language clinical NLP, specifically lung cancer named-entity recognition in French, remains methodologically nascent. The Tunisian healthcare system compounds this gap through documentation practices that blend standard French medical terminology with locally adapted clinical nomenclature, and through the near-total absence of publicly available annotated French clinical corpora in oncology. Existing tools developed and validated on English-language medical texts cannot be applied reliably in Francophone contexts without systematic adaptation [24].

Addressing these converging gaps in language, domain, and geographic representation is not merely a technical problem: it is a matter of health equity, as clinicians in French-speaking and North African settings are systematically excluded from the clinical AI pipeline benefiting English-language counterparts. Building on these identified deficits and leveraging the demonstrated effectiveness of transformer-based deep learning in clinical text processing, the present pilot study pursued three specific aims: (i) to benchmark BERT, RoBERTa, BioClinicalBERT, and CamemBERT on the RadGraph thoracic radiology dataset, establishing comparative performance baselines for clinical NER; (ii) to evaluate five transformer-based models, including DrBERT as a French biomedical specialist, on a newly constructed and manually annotated Tunisian lung cancer clinical corpus; and (iii) to demonstrate prototype deployment feasibility through a DrBERT-powered structured clinical interface enabling real-world decision support.

## 2. Materials and Methods

### 2.1. Ethical Approval

This study was conducted in accordance with the Declaration of Helsinki and current international guidelines for research practice in health and clinical informatics [25]. Clinical reports used in corpus construction were retrospectively collected and fully de-identified prior to any computational processing, in accordance with applicable national data protection legislation. The research protocol was reviewed and approved by the Ethics Committee of Hôpital Mami, Ariana, Tunisia (approval date: 30 June 2025). Following this approval, a retrospective extraction of 200 initial staging CT reports was performed from the hospital information system. The dataset consisted of retrospective clinical reports generated during routine patient care. The dataset included initial staging CT reports related to bronchopulmonary cancer cases retrospectively collected from the hospital database. Report selection aimed to ensure clinical and linguistic variability, including different clinical indications, imaging techniques, and tumor stages, in order to expose the NLP models to a broad spectrum of reporting patterns encountered in routine clinical practice. Inclusion criteria comprised initial staging CT reports related to suspected or confirmed lung cancer cases containing sufficient textual information for annotation and NLP. Duplicate or incomplete reports were excluded. The study was conducted under the scientific supervision of Dr. Saoussen Hantous-Zannad, Head of the Department of Medical Imaging at Hôpital Mami, and Prof. Hanene Boussi. The project was carried out in collaboration with the Higher Institute of Medical Technologies of Tunis (ISTMT) and the School of Public Health at the Université de Montréal, under the academic supervision of Prof. Jean-Noël Nikiema. Individual informed consent was waived by the institutional ethics committee due to the retrospective nature of the study and the exclusive use of fully anonymized clinical data. All reports were de-identified prior to transfer to the research team.

### 2.2. Baseline Dataset: RadGraph

The RadGraph dataset [30] comprises 600 annotated thoracic radiology reports reviewed and validated by board-certified radiologists. Annotations cover two primary entity categories: anatomy (ANAT) and observation (OBS). Observation entities are classified by certainty level into three subcategories—Definitely Present, Uncertain, and Definitely Absent—yielding four distinct entity labels for NER purposes: ANAT-DP, OBS-DP, OBS-U, and OBS-DA. The dataset was anonymized in compliance with the Health Insurance Portability and Accountability Act and serves as a publicly accessible benchmark for NLP method development in thoracic radiology.

In this study, RadGraph is used as an external benchmark dataset to evaluate the robustness and generalization capability of transformer-based NER models in a related clinical domain. Although RadGraph is not directly equivalent to the Tunisian corpus, it was selected because it consists of thoracic radiology reports, which are clinically related to lung cancer staging CT reports and share similar imaging-based descriptive patterns. Preprocessing pipelines for both datasets were aligned to ensure consistency in tokenization and formatting, while accounting for linguistic specificities.

However, important differences exist between the two datasets in terms of language (English vs. French), clinical context (general thoracic radiology vs. lung cancer staging reports), and annotation schema. Therefore, RadGraph is not intended as a direct benchmark for performance comparison with the Tunisian dataset, but rather as a complementary cross-domain evaluation to assess model generalization and robustness.

**Figure 1:**
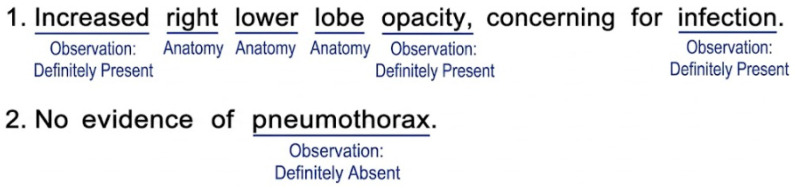
Excerpt from an annotated thoracic imaging report.

Figure 1 presents an excerpt from an annotated thoracic imaging report in the RadGraph dataset, illustrating the entity-labeling conventions applied throughout the benchmarking phase.

### 2.3. Data Preprocessing

Identical preprocessing was applied to both the RadGraph dataset and the Tunisian clinical corpus to ensure methodological consistency across the two study phases. Radiology and clinical reports frequently contain inconsistent spacing, special characters, and extraneous punctuation that disrupt tokenization reliability. Non-informative symbols and redundant whitespace were removed, and spacing was normalized across all tokens.

Each report was segmented into individual sentences and tokenized into words and meaningful symbols using the SpaCy tokenization library. Entities were encoded using the IOB2 (Inside–Outside–Beginning) tagging scheme, the standard annotation format for NER tasks in clinical informatics: B-ENTITY marks the beginning token of a named entity; I-ENTITY marks continuation tokens; O marks all tokens outside any named entity.

Preprocessed data were formatted according to the CoNLL standard, with each token on a separate line and its IOB2 label and sentences separated by blank lines.

**Figure 2:**
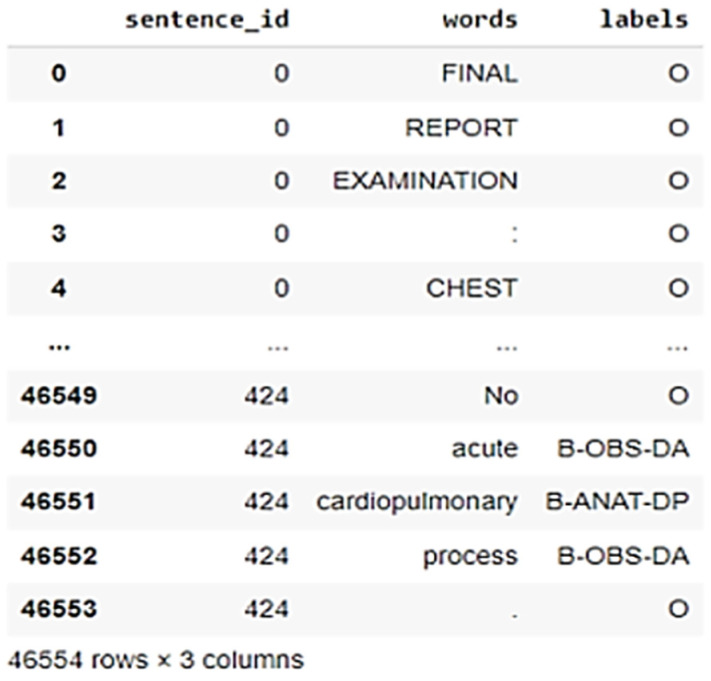
IOB2 labels Used in the RadGraph Dataset.

**Figure 3:**
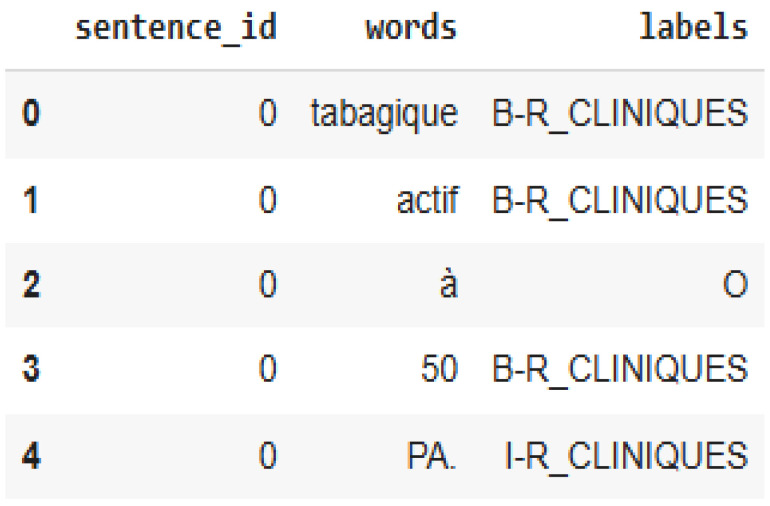
An excerpt from the Tunisian lung cancer corpus. Note: All data were fully anonymized to protect patient privacy. Abbreviations: PA (Pack-Years).

Figure 2 presents the IOB2 labels in the RadGraph dataset after preprocessing. Figure 3 shows a representative excerpt from the Tunisian lung cancer corpus after full preprocessing, illustrating the IOB2 encoding of the three annotated entity categories.

### 2.4. Construction and Annotation of the Tunisian Lung Cancer Corpus

Two hundred initial diagnostic reports were randomly selected from patients with confirmed lung cancer diagnoses at Mami Pneumo-Phthisiology Hospital, Ariana, Tunisia. Reports were manually de-identified prior to annotation, with all patient-identifying information removed or replaced with generic placeholders by trained clinical staff not involved in the annotation process.

A standard Tunisian initial diagnostic report contains four sections: Clinical Information, Techniques, Findings, and Conclusion. In this study, annotation was restricted to three clinically relevant sections: Clinical Information, Techniques, and Conclusion (TNM staging). The Findings section was excluded due to its heterogeneous and unstructured narrative content, which may introduce inconsistency in early-stage annotation design. This restriction was adopted to ensure a controlled and consistent benchmark corpus for structured information extraction in lung cancer reports.

The scope of this study is intentionally limited to the structured extraction of selected staging-related and clinically relevant information, rather than full clinical decision support or comprehensive oncology information extraction.

#### Annotation Scheme

Before initiating therapy, lung cancer patients undergo staging based on the TNM classification system. Three entity types were defined in consultation with oncology domain experts.

The entity types and their clinical scope are summarized in Table 1, which presents NER labels and their corresponding clinical information categories. Briefly:

- R_CLINIQUES captures the patient’s relevant clinical background, including smoking history, occupational exposures, and initial clinical investigations (fibroscopy, chest X-ray, CT scan) performed prior to or during the staging workup.
- TECHNIQUES covers technical details of imaging procedures conducted during the staging workup, including acquisition type, technical parameters, radiation dose, and scan coverage.
- STADE identifies the cancer stage exclusively through explicit TNM classification strings (e.g., T2N0M0) found within the report text.

This three-entity scheme was deliberately scoped to the subset of information most relevant to initial staging documentation. While this is sufficient for structured staging-related information extraction, we acknowledge that comprehensive oncology decision support requires a broader range of clinical variables. These include histological subtypes, molecular biomarkers (e.g., *EGFR*, *ALK*, or *PD-L1*), and performance status, which are not covered in the current annotation framework. The extension of this corpus to include such entities is considered future work to further enrich its clinical coverage.

Annotation was conducted using Med-Tator, a serverless text annotation tool designed for biomedical corpus development. Two annotators independently annotated each clinical report according to predefined annotation guidelines. The annotation process was primarily carried out by the first and last authors, both of whom have academic backgrounds in biomedical engineering and medical informatics.

Given the relatively focused scope of the annotation task, which mainly involved well-defined entities such as clinical stage, clinical findings, and technical information, the annotation guidelines were designed to ensure consistency and reproducibility across reports. All annotations were subsequently reviewed and validated by clinical experts from the Department of Medical Imaging at Hôpital Mami Ariana under the supervision of the department head. In cases of disagreement or uncertainty, annotations were discussed collaboratively until consensus was reached.

For a comprehensive breakdown of the annotation protocol, including specific inclusion/exclusion criteria, handling of negated clinical findings, and entity boundary rules, please refer to Supplementary Appendix B.

To assess annotation reliability, inter-annotator agreement was evaluated using both Cohen’s kappa coefficient and entity-level agreement metrics. The annotation process achieved a Cohen’s kappa score of 0.9565, indicating almost perfect agreement between annotators according to standard interpretation guidelines.

Entity-level agreement analysis further confirmed strong consistency in annotations, with a precision of 0.9621, recall of 0.9375, and F1-score of 0.9496. These metrics reflect the level of agreement between annotators at the entity level and demonstrate high consistency in span and label annotation decisions.

Although the dataset is relatively small and focused on a limited set of clinically well-defined entities, the combination of independent annotation followed by expert consensus review ensured the reliability and clinical validity of the final gold-standard corpus used in this study. Non-relevant sections of reports were excluded prior to model training to maintain annotation consistency and focus on the targeted information schema.

To provide a quantitative overview of the corpus composition, Table 2 summarizes the main statistical characteristics of the dataset, including the number of reports, sentences, and tokens, as well as the distribution of entity and non-entity tokens. This global overview allows for a better understanding of the linguistic structure and annotation density of the corpus prior to model training and evaluation.

These statistics provide a comprehensive overview of the corpus structure and highlight its intrinsic linguistic properties. In particular, they reveal a moderate imbalance between entity and non-entity tokens, which reflects the natural distribution of clinical narrative content in radiology reports. This distribution is not an annotation artifact but rather a consequence of the predominance of descriptive clinical information. These characteristics are important for interpreting model behavior and are further analyzed in the experimental results (Section 3). To further analyze the composition of the annotated dataset at a semantic level, Table 3 presents the distribution of entity types within the corpus. This breakdown allows us to assess the relative frequency of each clinical category and provides insight into potential class imbalance effects that may influence model learning and performance.

The entity-level distribution highlights a clear predominance of TECHNIQUES and R_CLINIQUES entities, while the STADE category remains comparatively underrepresented. This pattern reflects the intrinsic clinical structure of radiological reports, where descriptive findings and procedural information are more frequently documented than explicit staging information. The STADE category accounts for 6.33% of the annotated entities, which is consistent with its highly constrained semantic scope. Indeed, staging expressions are limited to standardized TNM classifications (e.g., T2N0, M1) and stage labels (e.g., Stage IV), which are inherently concise yet clinically critical for disease assessment and prognosis. In contrast, R_CLINIQUES and TECHNIQUES entities exhibit richer and more verbose linguistic realizations, which naturally dominate the narrative content of the corpus. Overall, this distribution reflects real-world clinical documentation practices and should be considered when interpreting model performance across entity types.

**Figure 4:**
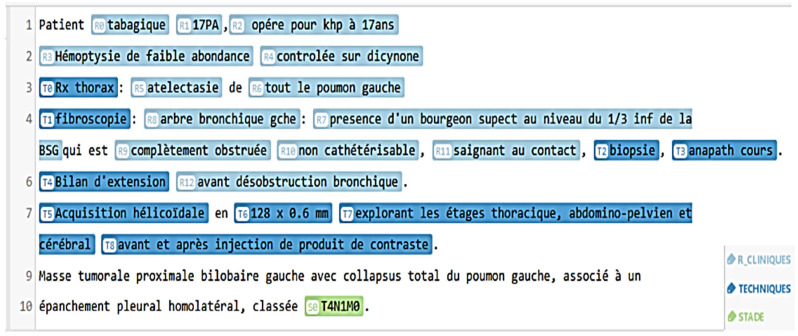
Sample annotated sentences from the lung cancer corpus. Note: Clinical identifiers have been removed or replaced for anonymization. Abbreviations: PA: Pack-Years (Paquets-Année); KHP: Pulmonary hydatid cyst (Kyste Hydatique Pulmonaire); BSG: Left Main Bronchus (Bronche Souche Gauche); anapath: Anatomic pathology (anatomopathologie).

Figure 4 presents sample annotated sentences from the Tunisian lung cancer corpus, illustrating the three entity categories in representative clinical text.

To further describe the dataset structure and ensure transparency in the experimental design, Table 4 reports the label distribution across the five-fold cross-validation splits of the Tunisian corpus.

Table 4 shows the label distribution across five folds of GroupKFold cross-validation on the Tunisian clinical NER corpus. The results show consistent preservation of all entity categories across folds, confirming stable stratified grouping and no label collapse during partitioning.

### 2.5. Model Selection and Architecture

#### 2.5.1. Benchmarking Phase (RadGraph Dataset)

Four pretrained transformer-based models were evaluated. BERT [24] is a general-purpose English language model pretrained on Wikipedia and BookCorpus through masked language modeling and next-sentence prediction objectives. RoBERTa is an optimized BERT variant trained with larger batch sizes, extended training duration, and removal of the next-sentence prediction objective, producing more robust contextual representations [27]. BioClinicalBERT [23] is initialized from BioBERT and further pretrained on MIMIC-III clinical notes, making it specifically suited to clinical free text. CamemBERT [25] is a French-language model pretrained on 138 GB of French text from the OSCAR corpus; its inclusion on the English RadGraph benchmark served as a methodological control, establishing a language-mismatch performance reference and justifying its subsequent evaluation on the French Tunisian corpus phase.

#### 2.5.2. Tunisian Corpus Phase

To evaluate the effectiveness of transformer-based architectures on the proposed Tunisian clinical corpus, we conducted a comprehensive comparison across five models, including general-purpose language models (BERT and RoBERTa), domain-adapted biomedical models (BioClinicalBERT and DrBERT), and CamemBERT, which was included as a French-language baseline despite the domain mismatch. Among these models, DrBERT [24], a state-of-the-art French biomedical language model based on the RoBERTa architecture and pretrained on the NACHOS French biomedical corpus, was selected as the primary candidate for the Tunisian corpus phase because it combines the architectural robustness of RoBERTa with domain-specific biomedical pretraining, making it particularly suitable for French clinical oncology text. Given the relatively limited size of the dataset, all models were evaluated using a stratified five-fold cross-validation strategy to ensure robust and reliable performance estimation while reducing variance across data partitions. In addition, a held-out test set evaluation was performed for each model to provide an additional reference for generalization performance.

### 2.6. Fine-Tuning Protocol

Each pretrained model was fine-tuned using a standard named-entity recognition (NER) pipeline based on the IOB2 tagging scheme. To ensure methodological rigor while respecting dataset-specific evaluation protocols, different experimental strategies were applied across the two study phases.

For the RadGraph benchmarking phase, experiments were conducted using the official dataset release containing a total of 600 annotated thoracic radiology reports, which natively provides a standardized 80/10/10 train–validation–test partition. In contrast, for the Tunisian clinical corpus, a stratified five-fold cross-validation strategy was adopted due to the limited dataset size, in order to improve robustness and provide statistically reliable performance estimates. Models were fine-tuned using a maximum of 10 epochs for RadGraph and 5 epochs for the Tunisian corpus, with learning rates of 5 × 10^−5^ and 2 × 10^−5^ respectively, and batch sizes of 16 and 8. Early stopping based on validation F1-score was applied to prevent overfitting in both settings. Despite differences in evaluation protocols, all models within each dataset phase were trained under a unified hyperparameter configuration to ensure fair intra-phase comparison. This design isolates the impact of model architecture and pretraining data while maintaining controlled experimental conditions across all experiments. All results are reported using entity-level precision, recall, and F1-score, with mean ± standard deviation computed over cross-validation folds for the Tunisian corpus. A fixed random seed (42) was used across Python, NumPy, and PyTorch to ensure full reproducibility. Models were initialized with their native pretrained tokenizers and optimized using the AdamW optimizer with default parameters. The experiments were implemented in Python 3.10 using PyTorch (version 2.x) and the Hugging Face Transformers library (version 4.36.0), within the SimpleTransformers NER framework. The following pretrained checkpoints were used for fine-tuning: bert-base-multilingual-cased for BERT, roberta-base for RoBERTa, emilyalsentzer/Bio_ClinicalBERT for BioClinicalBERT, camembert-base for CamemBERT, and Dr-BERT/DrBERT-7GB for DrBERT. All experiments were executed on NVIDIA GPU hardware with CUDA acceleration.

### 2.7. Evaluation Metrics

Final performance was assessed on the held-out test set using precision, recall, and F1-score computed at the entity boundary level. Precision measures the proportion of predicted entities that are correct (TP/(TP + FP)). Recall measures the proportion of true entities retrieved (TP/(TP + FN)). The F1-score is the harmonic mean of precision and recall, providing a single balanced performance index.

Evaluation loss was recorded as an additional indicator of model convergence and optimization stability.

### 2.8. Prototype Development

The best-performing model on the Tunisian corpus was embedded in a prototype clinical interface designed to assess the feasibility of real-world deployment. Visualization and exploratory analyses were conducted using Power BI. The prototype generates structured patient summaries providing immediate access to extracted clinical entities—prior conditions, technical modalities, and TNM staging—alongside navigable access to the full original report within a unified dashboard environment.

### 2.9. AI Usage Statement

In preparing this manuscript, the authors used Claude (Anthropic) to improve the clarity and grammatical correctness of selected passages. The tool was used to revise text for an enhanced academic tone, check for grammatical errors, and improve the quality of the English language. The authors did not use AI for data analysis, interpretation, or generation of scientific content. After using this tool, the authors thoroughly reviewed and edited all content and took full responsibility for the accuracy, integrity, and scientific validity of the work [27,28].

## 3. Results

### 3.1. Baseline Performance on the RadGraph Dataset

The performance metrics for the four transformer-based models evaluated on the RadGraph test set are summarized in Table 5, establishing a comparative reference for the study. RoBERTa achieved the highest overall performance, recording a precision of 0.869, a recall of 0.877, an F1-score of 0.873, and an evaluation loss of 0.275. BioClinicalBERT performed closely behind with a precision of 0.858, a recall of 0.878, an F1-score of 0.868, and an evaluation loss of 0.254, the lowest across all models. BERT achieved a precision of 0.855, a recall of 0.859, an F1-score of 0.857, and an evaluation loss of 0.441. CamemBERT recorded a precision of 0.670, a recall of 0.695, an F1-score of 0.682, and an evaluation loss of 0.529. The performance differential between CamemBERT and the three English-pretrained models (delta F1: 0.175–0.191) reflects the language mismatch between its French pretraining corpus and the English RadGraph evaluation context, confirming that linguistic alignment is the primary driver of performance differences in this benchmarking phase. Based on these results, RoBERTa was selected for direct transfer to the Tunisian corpus phase.

### 3.2. Performance Evaluation on the Tunisian Clinical Corpus

#### 3.2.1. Corpus Comparison of Cross-Validation and Test Set Performance Across Models

To ensure statistical robustness given the limited dataset size, a five-fold GroupKFold cross-validation strategy was applied. Performance is measured using entity-level precision, recall, and F1-score, reported as mean ± standard deviation across cross-validation folds, along with corresponding results on the held-out test set. Table 6 summarizes the comparison of cross-validation and test set performance across all models. Additionally, Appendix A provides the detailed five-fold cross-validation results for each model.

Table 6 presents the comparative performance metrics of the evaluated transformer-based models across both the five-fold cross-validation (CV) phase and the independent hold-out test set. The empirical results reveal distinct performance profiles across the different architectures.

During the five-fold cross-validation phase, the general multilingual model (bert-base-multilingual-cased) achieved the highest overall metrics, yielding a mean F1-score of 0.843 ± 0.005 (precision: 0.831, recall: 0.856). DR-BERT followed closely, with a cross-validation mean F1-score of 0.832 ± 0.010 (precision: 0.820, recall: 0.846).

However, when evaluated on the independent hold-out test set, DR-BERT demonstrated superior generalization capabilities, achieving the highest F1-score of 0.824, with balanced precision and recall values of 0.819 and 0.828, respectively. The baseline RoBERTa model maintained competitive performance, obtaining a test-set F1-score of 0.791 (precision: 0.773, recall: 0.810). In contrast, BioClinicalBERT and CamemBERT showed lower generalization performance on the test set, with F1-scores of 0.775 (precision: 0.751, recall: 0.801) and 0.788 (precision: 0.774, recall: 0.802), respectively.

Notably, CamemBERT exhibited an exceptionally high evaluation loss of 5.340 on the test set, compared to the relatively stable loss values (ranging from 0.333 to 0.388) observed across all other models. The relative consistency between cross-validation and test set results for most models further underscores stable behavior across different data splits.

Overall, these findings highlight the critical importance of domain-specific pre-training strategies in enhancing model robustness on unseen data.

#### 3.2.2. Error Analysis

To evaluate model performance beyond aggregate metrics, a qualitative error analysis was conducted. Errors were categorized as follows: false positives (FP), where the model predicts an entity that does not exist; false negatives (FN), where a true entity is missed; and boundary errors, where the correct entity type is detected, but BIO tagging or span delimitation is incorrect. These errors mainly reflect segmentation difficulties in the telegraphic structure of clinical narratives.

The quantitative distribution of these errors is summarized in Table 7, highlighting consistent patterns across models.

The analysis reveals three main findings:

Boundary errors are the most frequent issue, particularly for complex medical expressions in radiology reports;

CamemBERT exhibits a precision-oriented behavior with fewer false positives but higher false negatives, indicating a trade-off between precision and recall;

BioClinicalBERT shows a higher overall error rate, likely due to domain and language mismatch with the French/Tunisian clinical corpus.

### 3.3. Prototype Deployment and Clinical Interface

**Figure 5:**
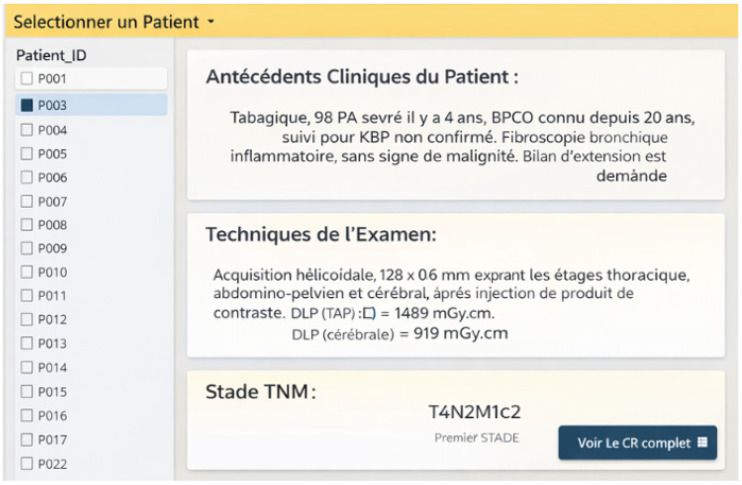
Patient Profile View—Interface 1. Patient Profile View—Interface 1. *Note: The user interface is natively in French, as designed for local clinical workflows. The main panel headers translate to English as follows: "Antécédents Cliniques du Patient" (Patient Clinical History), "Techniques de l’Examen" (Examination Techniques), and "Stade TNM" (TNM Stage)*.

**Figure 6:**
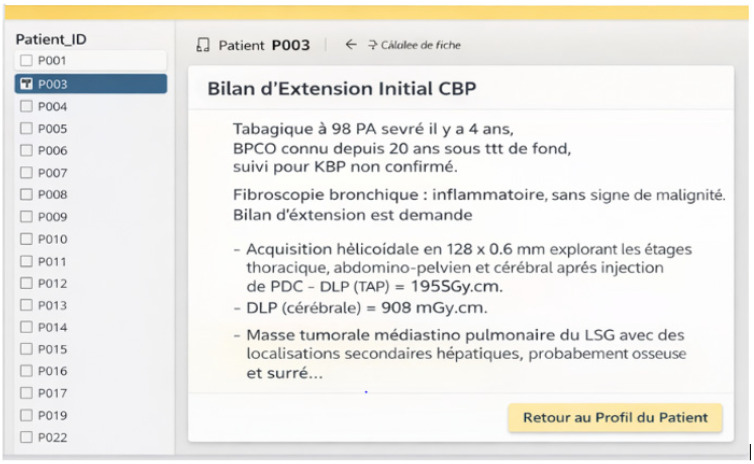
Detailed Report Access—Interface 2. *Note: The application interface displays raw, anonymized clinical text natively in French. Key interface navigation terms translate to English as: "Bilan d’Extension Initial CBP" (Initial Lung Cancer Staging Assessment) and "Retour au Profil du Patient" (Return to Patient Profile)*.

DrBERT was selected for prototype deployment due to its superior precision on the Tunisian corpus. Figure 5 presents the Patient Profile View (Interface 1), which provides a structured clinical summary of extracted entities, including prior conditions, imaging modalities, and TNM staging information derived from the unstructured initial diagnostic report. Figure 6 shows the Detailed Report Access (Interface 2), offering navigable access to the complete original clinical text alongside DrBERT’s structured output within a unified Power BI dashboard.

The prototype successfully transforms unstructured initial lung cancer diagnostic reports into clinician-accessible structured summaries, illustrating the primary operational objective of this study. It is important to note that this interface represents a conceptual demonstration of the system; formal usability testing with clinicians has not yet been conducted. Future work will involve a structured usability assessment using validated instruments, such as the System Usability Scale (SUS), to quantitatively evaluate the interface’s effectiveness and user satisfaction.

## 4. Discussion

This pilot study demonstrates that transformer-based NER models can effectively extract structured clinical entities from unstructured French-language Tunisian lung cancer diagnostic reports, achieving performance levels comparable to established English-domain benchmarks. In the preliminary phase, comparative performance baselines on the RadGraph dataset (English thoracic benchmark) showed that RoBERTa achieved the highest F1-score of 0.873, followed closely by BioClinicalBERT (0.868) and BERT (0.857). Conversely, CamemBERT recorded a significantly lower score of 0.682, a predictable consequence of the linguistic mismatch between French pretraining and English evaluation data. On the locally constructed Tunisian corpus, DR-BERT outperformed the baseline models on the independent test set, achieving a test-set F1-score of 0.824 (compared to 0.791 for RoBERTa). These findings confirm that language-specific biomedical pretraining is a critical driver of robust generalization in non-English specialized clinical contexts, enabling the model to effectively capture the nuances of French oncology documentation.

### 4.1. Comparative Performance Baseline on the RadGraph Dataset

RoBERTa’s F1-score of 0.873 on the RadGraph test set, with BioClinicalBERT at 0.868 and BERT at 0.857, reflects the transformer performance hierarchy established in the clinical NLP literature. The near-identical performance of RoBERTa and BioClinicalBERT (delta F1: 0.005) on a thoracic radiology benchmark is consistent with the well-documented finding that general English optimization in RoBERTa’s training regime—larger batch sizes, extended training, removal of next-sentence prediction—produces representations competitive with domain-specific pretraining on general clinical entity recognition benchmarks [30,31]. BioClinicalBERT’s lower evaluation loss compared to RoBERTa (0.254 vs. 0.275) despite marginally lower F1 suggests superior probability calibration, a property of potential clinical relevance in deployment contexts where model uncertainty must be communicated to end users [23]. Abdaoui et al., conducting the most methodologically comparable study available in the Tunisian clinical NLP context, achieved F1 0.97 for entity extraction from Tunisian pathology reports using a hybrid BioClinicalBERT model augmented with dense retrieval and validated on 560 reports [28]. The performance gap between that study (0.97) and the present benchmarking phase (0.873) is attributable to differences in the datasets: Abdaoui et al. used a larger corpus, a more granular annotation scheme, and a hybrid retrieval-augmented architecture. CamemBERT’s F1 of 0.682 on the English RadGraph dataset is not architecturally informative; its French-corpus pretraining renders it predictably suboptimal on English clinical text, and its inclusion in the benchmarking phase served exclusively to establish a language-mismatch baseline [25]. These findings indicate that clinical NLP practitioners selecting models for English thoracic radiology NER should prioritize RoBERTa or domain-pretrained English variants over general multilingual architectures.

### 4.2. Performance on the Tunisian Corpus

The experimental results validate that while general-purpose multilingual architectures provide strong baselines, language- and domain-specific pretraining serves as the key driver of robust model generalization in specialized non-English clinical contexts.

The contrasting behavior between the cross-validation phase and the hold-out test set offers critical insights into model stability. The strong performance of the multilingual BERT (mBERT) baseline during cross-validation (mean F1: 0.843 ± 0.005) indicates that its massive multilingual pretraining allows it to capture dominant structural and syntactic patterns present within the training splits effectively. However, its subsequent performance drop on the independent test set (F1: 0.816) suggests a higher degree of variance. Conversely, DR-BERT maintained highly stable metrics from cross-validation (0.832) to the test set (0.824). This behavior confirms that domain-specific pretraining on French medical corpora (NACHOS) builds more resilient representations that prevent catastrophic drop-offs when encountering entirely unseen clinical reports.

The importance of domain alignment is further emphasized by the limitations observed when general language models are applied to specialized clinical text. CamemBERT, despite being trained on native French, lacks biomedical specialization. This language-domain mismatch resulted in a lower test F1-score (0.788) relative to DR-BERT and was accompanied by an exceptionally high evaluation loss (5.340). This extreme loss value strongly points to optimization and convergence difficulties, highlighting how a model trained on general text struggles to confidently evaluate the dense, highly technical vocabulary inherent to oncology documentation.

Interestingly, a cross-lingual domain transfer was observed with Bio-ClinicalBERT. Despite being pretrained exclusively on English clinical data, it achieved a respectable cross-validation score (0.821) and maintained a solid test-set recall of 0.801. This phenomenon is likely explained by the high density of shared, Latin-derived medical terminology common to both English and French oncology vocabularies. Nevertheless, its lower test set precision (0.751) and overall F1-score (0.775) underscore that cross-lingual transfer cannot fully substitute for target-language-specific pretraining when navigating local syntax and structural report constraints.

Ultimately, DR-BERT’s balanced precision (0.819) and recall (0.828) on the hold-out set confirm its readiness for downstream clinical decision support applications. In oncology data management, minimizing missing information (ensuring a high recall) while maintaining strict diagnostic certainty (high precision) is vital, establishing this specialized architecture as a foundational infrastructure for French-language clinical NLP tasks in Tunisia and comparable Francophone settings.

### 4.3. Clinical Relevance of the Prototype

The DrBERT-powered prototype interface successfully transformed unstructured initial lung cancer diagnostic reports into structured, navigable clinical summaries covering prior conditions, imaging modalities, and TNM staging. This proof-of-concept directly addresses the operational challenge motivating the study: converting the unstructured clinical narrative into a format compatible with large-scale data analysis, cohort construction, and real-world evidence generation. Machine learning applications in healthcare have demonstrated that automated extraction of structured data from clinical text supports patient stratification, risk prediction, and clinical workflow optimization [32]. The Power BI visualization layer embedded in the prototype provides a low-friction integration pathway accessible to clinicians without computational expertise, consistent with the principle established across clinical AI implementation research that adoption depends on reducing rather than increasing cognitive burden [17]. The present prototype represents the first operational instantiation of this principle in the Tunisian lung cancer documentation context, laying the groundwork for systematic clinical validation in a subsequent study phase with expanded corpus coverage and formal usability assessment.

### 4.4. Positioning Within Global Clinical NLP

The present study addresses a geographic and linguistic gap that clinical NLP research has systematically left unresolved. The near-exclusive focus of clinical NLP development on English-language corpora has created a technological disparity that mirrors and potentially amplifies existing inequities in global health research capacity. Francophone African healthcare systems, including Tunisia’s, have been largely absent from the clinical AI development pipeline, despite the fact that French-language clinical documentation poses NLP challenges distinct from those of English and other Romance languages. The construction of a 200-report annotated Tunisian lung cancer corpus in the present study addresses this deficit at its source, creating an annotated clinical resource that did not previously exist. Abdaoui et al.’s parallel construction of a Tunisian pathology NLP corpus confirms that this approach is tractable and extensible across clinical domains [23]. This work is also situated within the broader concern about appropriate AI deployment in clinical reasoning contexts. Dergaa et al. have characterized a pattern of professional reasoning degradation resulting from the uncritical acceptance of AI outputs as a substantive risk of overreliance on general-purpose AI systems [22]. The present study’s design, which embeds domain-adapted NER into interfaces that present structured outputs alongside original reports for clinician review, precisely mitigates this risk: AI augments clinical annotation without supplanting human judgment.

### 4.5. Limitations

This study presents several methodological and practical limitations that should be considered when interpreting the results and their potential for generalization. First, although the proposed models were evaluated using standard performance metrics (precision, recall, and F1-score) together with cross-validation and held-out test sets, the study remains constrained by the size and origin of the Tunisian corpus. The dataset comprises 200 clinical reports collected from a single institution, which may limit data diversity and influence the generalizability of the findings. In addition, the dataset exhibits a moderate class imbalance, with TECHNIQUES and R_CLINIQUES entities being more frequent than STADE entities. While this distribution reflects the natural structure of radiology reports, it may still influence model performance, particularly for underrepresented classes. Second, although cross-validation was performed to assess model robustness across different data splits, the relatively small corpus size remains a limiting factor for deep learning-based clinical NLP tasks. Nevertheless, the stable performance observed across folds and the held-out test set suggests promising robustness within the studied context. Future research should investigate the scalability of the proposed framework on larger and more heterogeneous clinical datasets to further assess its adaptability across diverse healthcare environments. Third, inter-annotator agreement was evaluated during corpus construction using Cohen’s Kappa, ensuring a satisfactory level of annotation consistency. However, this agreement was assessed within a single institutional and annotator setting. Future work could extend inter-annotator agreement evaluation through collaborations involving multiple annotation teams and broader clinical expertise in order to further strengthen the robustness and external validity of the annotation guidelines and gold standard corpora. Fourth, the annotation schema adopted in this study focuses primarily on staging-oriented clinical information, including smoking history, medical history, imaging techniques, acquisition parameters, and TNM classification. While this enables structured extraction of clinically relevant entities for lung cancer staging, it does not yet cover broader oncological dimensions such as histological subtypes, molecular biomarkers (e.g., *EGFR*, *ALK*, *PD-L1*), treatment strategies, or patient performance status. This limits the current scope to staging-related information, and future extensions should aim to incorporate these additional clinical dimensions to enable a more comprehensive representation of lung cancer patient records. Fifth, although the evaluation framework includes full metric reporting and cross-validation, the study does not include external validation on independent datasets. Therefore, while the results demonstrate strong performance on the Tunisian corpus, they should currently be interpreted within the context of the studied clinical environment. Future investigations should evaluate the proposed models across broader clinical contexts and heterogeneous healthcare infrastructures to further examine their generalizability and robustness. Sixth, the prototype system developed in this study serves as a proof-of-concept for integrating structured NLP outputs into a clinician-oriented interface. However, it has not yet undergone formal usability testing or clinical workflow evaluation. Consequently, its clinical usability, adoption potential, and integration into routine healthcare practice remain to be validated through structured user studies and real-world deployment scenarios.

Finally, despite promising results, real-world deployment in Tunisian healthcare settings faces significant regulatory, privacy, and interoperability challenges. Clinical data access is strictly regulated by institutional ethics committees and national data protection frameworks, requiring formal approval and limiting large-scale data availability. Although anonymization procedures are applied, residual identifiers may still pose a theoretical risk of re-identification when applying advanced NLP techniques. Furthermore, the heterogeneity and partial digitization of Electronic Health Record (EHR) systems, combined with limited interoperability standards such as HL7 or FHIR, complicate the seamless integration of NLP-based tools into clinical workflows. These factors highlight the need for future work focusing on privacy-preserving methods and interoperable AI systems adapted to local healthcare infrastructures.

## 5. Conclusions

This pilot benchmarking and fine-tuning study evaluated transformer-based named-entity recognition models for automated clinical annotation of French-language lung cancer diagnostic reports from a Tunisian hospital, using a two-phase design that first established comparative performance baselines on the publicly available RadGraph English thoracic radiology benchmark and then evaluated all five models on a newly constructed and manually annotated Tunisian lung cancer clinical corpus of 200 initial diagnostic reports. On the RadGraph dataset, RoBERTa achieved the highest F1-score of 0.873, outperforming BioClinicalBERT (0.868), BERT (0.857), and CamemBERT (0.682). CamemBERT’s lower performance is attributable to its French pretraining applied to an English evaluation dataset. On the Tunisian lung cancer corpus, DR-BERT achieved the highest performance with a test-set F1-score of 0.824, outperforming RoBERTa (0.791). This confirms that biomedical pretraining in the target clinical language is the primary driver of performance in specialized French oncology text. The DR-BERT-powered prototype interface successfully generated structured clinical summaries encompassing prior conditions, imaging modalities, and TNM staging from unstructured diagnostic reports, establishing the operational feasibility of NLP-driven clinical annotation in this context. These results carry direct implications for clinical data infrastructure development in Tunisia and comparable Francophone and resource-limited healthcare systems: French biomedical models should be systematically prioritized over English-domain or general multilingual alternatives when processing French clinical text, and local corpus annotation must be treated as the foundational institutional investment enabling NLP pipeline calibration to local documentation practices. Broader progress toward comprehensive lung cancer data management in Tunisia depends on the establishment of larger, standardized, and interoperable clinical databases—an objective the present study both motivates and technically supports. Construction of this infrastructure will enable future models to extract the full clinical entity repertoire required for precision oncology and ultimately contribute to a clinically actionable, AI-supported oncology data ecosystem serving the Francophone world.

## Figures and Tables

**Table 1:** NER labels and clinical information categories.

Groups	Label NER	What Does It Include
Clinical Information	R_CLINIQUES	A Smoker? Number of packs/years Professional Exposure Medical History
Techniques	TECHNIQUES	Acquisition Type Technical Parameters Anatomical Region Explored Contrast Phase Dose Irradiation Dose Dose Explored Area
Conclusion	STADE	Explicit TNM classification codes

**Table 2:** Statistical characteristics of the lung cancer Tunisian corpus.

Metric	Reports	Sentences	Tokens	Average Sentence Length	Entity Tokens	Non-Entity Tokens (O)	Total Annotated Entities
Value	200	2046	19,918	9.74	69.01%	30.99%	3663

**Table 3:** Entity distribution in the Tunisian lung cancer corpus.

Entity Type	Count	Percentage
R_CLINIQUES	1615	44.09%
TECHNIQUES	1816	49.58%
STADE	232	6.33%
Total	3663	100%

**Table 4:** Label distribution across five-fold cross-validation (Tunisian clinical corpus).

Label	Fold 1	Fold 2	Fold 3	Fold 4	Fold 5
O	1140	1147	1251	1178	1156
I-TECHNIQUES	1336	1365	1301	1351	1330
I-R_CLINIQUES	493	476	457	452	517
B-TECHNIQUES	362	370	348	360	376
B-R_CLINIQUES	342	322	313	333	305
B-STADE	44	44	53	47	44
I-STADE	21	14	14	16	9

**Table 5:** Detailed baseline performance metrics on the RadGraph dataset.

Model	Precision	Recall	F1-Score	Evaluation Loss
RoBERTa	0.869	0.877	0.873	0.275
BioClinicalBERT	0.858	0.878	0.868	0.254
BERT	0.855	0.859	0.857	0.441
CamemBERT	0.670	0.695	0.682	0.529

Note: Evaluation conducted on the RadGraph test set (10% of 600 annotated thoracic radiology reports). F1-score computed at the entity level using the IOB2 annotation scheme. CamemBERT performance is expected to be lower given the language mismatch between the French pretraining corpus and the English evaluation dataset.

**Table 6:** Comparison of cross-validation and test set performance across all models.

Model	Setting	Precision	Recall	F1-Score (Mean ± Std)	Loss	Notes
DR-BERT	5-fold CV	0.820	0.846	0.832 ± 0.010	—	French biomedical pretraining (NACHOS)
	Test set	0.819	0.828	0.824	0.340	
RoBERTa	5-fold CV	0.799	0.826	0.812 ± 0.019	—	Best RadGraph performer
	Test set	0.773	0.810	0.791	0.333	
BioClinicalBERT	5-fold CV	0.803	0.839	0.821 ± 0.005	—	Clinical domain; English pretrained
	Test set	0.751	0.801	0.775	0.388	
CamemBERT	5-fold CV	0.795	0.795	0.795 ± 0.023	—	General French; not biomedical
	Test set	0.774	0.802	0.788	5.340	
BERT	5-fold CV	0.831	0.856	0.843 ± 0.005	—	General multilingual model (bert-base-multilingual-cased)
	Test set	0.804	0.828	0.816	0.356	

**Table 7:** Quantitative distribution of model errors.

Model	FP	FN	Boundary	Total
CamemBERT	76	118	60	254
DrBERT	84	93	70	247
BERT (m-base)	90	83	72	245
RoBERTa	104	104	69	277
BioClinicalBERT	111	94	78	283

## Data Availability

Publicly available datasets were analyzed in this study. The RadGraph dataset was obtained from PhysioNet and is available at: https://physionet.org/content/radgraph/1.0.0/ (accessed on 15 May 2025). The Tunisian clinical corpus used in this study is not publicly available due to patient privacy and institutional ethical restrictions. For academic and non-commercial research purposes, access can be granted upon reasonable request, subject to approval by the data governance committee and the signing of a data usage agreement. Requests should be directed to: hanene.boussi@istmt.utm.tn and ranim.yahyaoui@etudiant-istmt.utm.tn.

## Appendix A. Five-Fold Cross-Validation Results

**Table A1:** DrBERT cross-validation results.

Fold	Precision	Recall	F1-Score	Evaluation Loss
Fold 1	0.8171	0.8302	0.8236	0.3731
Fold 2	0.8023	0.8383	0.8199	0.3574
Fold 3	0.8156	0.8487	0.8318	0.3702
Fold 4	0.8368	0.8595	0.8480	0.3931
Fold 5	0.8262	0.8524	0.8391	0.3268
Mean ± SD	0.820	0.846	0.832 ± 0.010	—

Table A1 shows the five-fold cross-validation performance of the DrBERT model on the Tunisian clinical corpus using entity-level evaluation metrics. Results are reported as mean ± standard deviation across folds.

**Table A2:** RoBERTa cross-validation results.

Fold	Precision	Recall	F1-Score	Evaluation Loss
Fold 1	0.776	0.806	0.791	0.349
Fold 2	0.775	0.811	0.793	0.285
Fold 3	0.792	0.833	0.812	0.301
Fold 4	0.833	0.842	0.837	0.333
Fold 5	0.819	0.837	0.828	0.327
Mean ± SD	0.799	0.826	0.812 ± 0.019	—

Table A2 shows the five-fold cross-validation performance of the RoBERTa model on the Tunisian clinical corpus using entity-level evaluation metrics. Results are reported as mean ± standard deviation across folds.

**Table A3:** BioClinicalBERT cross-validation results on the Tunisian clinical corpus.

Fold	Precision	Recall	F1-Score	Evaluation Loss
Fold 1	0.796	0.836	0.815	0.384
Fold 2	0.805	0.837	0.821	0.294
Fold 3	0.798	0.836	0.817	0.332
Fold 4	0.814	0.843	0.828	0.355
Fold 5	0.804	0.841	0.822	0.349
Mean ± SD	0.803	0.839	0.821 ± 0.005	—

Table A3 shows the five-fold cross-validation performance of the BioClinicalBERT model on the Tunisian clinical corpus using entity-level evaluation metrics. Results are reported as mean ± standard deviation across folds.

**Table A4:** BERT cross-validation results on the Tunisian clinical corpus.

Fold	Precision	Recall	F1-Score	Evaluation Loss
Fold 1	0.833	0.841	0.837	0.341
Fold 2	0.829	0.863	0.846	0.295
Fold 3	0.821	0.857	0.839	0.343
Fold 4	0.835	0.853	0.844	0.374
Fold 5	0.835	0.868	0.851	0.336
Mean ± SD	0.831	0.856	0.843 ± 0.005	—

Table A4 shows the five-fold cross-validation performance of the BERT model on the Tunisian clinical corpus using entity-level evaluation metrics. Results are reported as mean ± standard deviation across folds.

**Table A5:** CamemBERT cross-validation results on the Tunisian clinical corpus.

Fold	Precision	Recall	F1-Score	Evaluation Loss
Fold 1	0.747	0.765	0.756	5.340
Fold 2	0.804	0.781	0.793	5.477
Fold 3	0.818	0.831	0.824	5.334
Fold 4	0.815	0.772	0.793	5.208
Fold 5	0.793	0.826	0.809	5.482
Mean ± SD	0.795	0.795	0.795 ± 0.023	—

Table A5 shows the five-fold cross-validation performance of the CamemBERT model on the Tunisian clinical corpus using entity-level evaluation metrics. Results are reported as mean ± standard deviation across folds.

## Appendix B. Detailed Annotation Guidelines

### Appendix B.1. Overview

To ensure corpus reproducibility and inter-annotator consistency, this protocol defines the boundaries and criteria for labeling entities in Tunisian lung cancer diagnostic reports. A span-level annotation approach was adopted, where clinical modifiers (adjectives, quantities, and technical parameters) are included within the entity boundary.

### Appendix B.2. Entity Inclusion and Exclusion Criteria

**Table:**

Entity Label	Definition	Inclusion Criteria	Exclusion Criteria
R_CLINIQUES	Patient-specific Medical Background and risk factors	Smoking status and quantitative history (tabagique, 17PA), surgical history (opéré pour khp), initial clinical symptoms (Hémoptysie), and associated medical management (controlée sur dicynone).	Pure anatomical descriptions or morphological observations of the tumor (e.g., “bilobar mass”) found in the findings section
TECHNIQUES	Technical specifications of the imaging or diagnostic procedure	Imaging modalities (Rx thorax, fibroscopie), precise technical parameters (128 × 0.6 mm), acquisition modes (hélicoïdale), contrast media, and sampling procedures (biopsie, anapath)	The results or findings derived from these techniques (e.g., “presence of a bud” is an observation/finding, not a technique)
STADE	Explicit staging based on the TNM classification system	Strictly limited to the Standardized alphanumeric TNM classification codes (T4N1M0) usually located in the conclusion	Introductory terms (e.g., “classified as”, “stage”) or narrative descriptions of tumor extension (e.g., “pleural effusion”)

### Appendix B.3. Linguistic Rules and Edge Case Handling

To standardize the extraction of high-value clinical data, the following rules were applied across all 200 reports:

- Granularity of Metrics: Numerical values and units (e.g., 17PA, 128 × 0.6 mm) are systematically included within the label to preserve the clinical significance of the data.
- Modifiers and Qualifiers: Adjectives qualifying a technique (e.g., hélicoïdale) or a clinical state (e.g., faible abondance) are integrated into the annotated span.
- Separation of Procedural Steps: Multi-step procedures are isolated into distinct entities. For example, biopsie and anapath are labeled as two separate technical entities, even when occurring during a single fibroscopy.
- Contiguous Spans: Compound expressions (e.g., Acquisition hélicoïdale, injection de produit de contraste) are annotated as single, unbroken blocks to facilitate structured information extraction.
- Negation: When a clinical sign or risk factor is negated, the negation marker is included in the span (e.g., non cathétérisable) to maintain the semantic truth of the report.
